# General practice attendances among patients attending a post-COVID-19 clinic: a pilot study

**DOI:** 10.3399/BJGPO.2021.0016

**Published:** 2021-05-12

**Authors:** John Broughan, Geoff McCombe, Gordana Avramovic, Des Crowley, Cheyenne Downey, Joanne Downey O'Sullivan, Ronan Fawsitt, Tina McHugh, Eileen O'Connor, Carla Perrotta, Aoife G Cotter, John S Lambert, Walter Cullen

**Affiliations:** 1 School of Medicine, University College Dublin, Dublin, Ireland; 2 Mater Misericordiae University Hospital, Dublin, Ireland; 3 Clinical Research Centre, University College Dublin, Dublin, Ireland; 4 Castle Gardens Surgery, Kilkenny, Ireland; 5 Ireland East Hospital Group, Dublin, Ireland; 6 School of Public Health, Physiotherapy and Sports Sciences, University College Dublin, Dublin, Ireland; 7 Centre for Experimental Pathogen Host Research, University College Dublin, Dublin, Ireland

**Keywords:** COVID-19, COVID-19 virus, SARS-CoV-2, follow-up studies, general practice, long-term care, pilot projects

## Abstract

**Background:**

About 10–35% of people with COVID-19 need medical care within 3 weeks of infection. However, the prevalence of ongoing care needs among those experiencing severe COVID-19 illness is unclear.

**Aim:**

This pilot study aimed to address this knowledge gap by examining GP attendance trends among patients attending a post-COVID-19 hospital follow-up clinic, 3–6 months after an initial clinic visit.

**Design & setting:**

Data were collected from adult patients attending a post-COVID-19 follow-up clinic at the Mater Misericordiae University Hospital (MMUH), Dublin, Ireland.

**Method:**

Participants completed questionnaires outlining their demographics; medical histories; emergency hospital admissions and readmissions where applicable; and, where relevant, GP attendances following hospital discharge. Analyses were conducted using descriptive and inferential statistics.

**Results:**

Participants’ (*n* = 153) median age was 43.5 years (interquartile range [IQR] = 30.9–52.1 years). There were 105 females (68.6%, 95% confidence interval [CI] = 61.3% to 75.9%). Various medical histories were reported among participants. Sixty-seven (43.2%, 95% CI = 35.9% to 51.6%) received emergency COVID-19 hospital care. Older adults, males, intensive care unit [ICU] admissions, and readmissions were common among hospital attendees. Of the hospital attendees, 16 (24%, 95% CI = 13.7% to 34.2%) attended GPs within 7 days of hospital discharge, and 26 (39%, 95% CI = 27.3% to 50.7%) within 30 days. Older adults, people with pre-existing medical conditions, and individuals admitted to ICU and/or readmitted to hospital were common among general practice attendees.

**Conclusion:**

Persistent health issues appear to be common among patients with severe COVID-19, particularly those who are older adults, have pre-existing health problems, and who had been in ICU and/or readmission care. Larger scale studies of ongoing COVID-19 care needs in primary care and general practice are required.

## How this fits in

This pilot study advances existing knowledge by highlighting the prevalence of the ongoing care needs of patients with severe COVID-19. The findings suggest that 39% (95% CI = 27.3 to 50.7%) of patients with severe COVID-19 require care within 30 days, the kind that is routinely provided by GPs. The findings also suggest that long-term care needs may be greater among older adults, patients with pre-existing health conditions, and patients requiring ICU and/or readmission care. Larger-scale studies in primary care and general practice are required to obtain more precise estimates of persistent illness among severe COVID-19 cases in communities.

## Introduction

As of late January 2021, there have been 98 925 221 cases of COVID-19 globally,^1^ with 188 923 cases in Ireland.^2^ Worldwide, 2 127 294 people have died with COVID-19 infection,^1^ and 2977 people have died with COVID-19 in Ireland.^2^ People of all ages can contract COVID-19,^3^ but those aged <50 years are more likely to do so.^3^ In contrast, individuals aged >50 years are more likely to die because of COVID-19 infection.^3^ Both males and females have similar chances of being infected with COVID-19,^4,5^ and in Ireland, COVID-19 death rates are similar for both sexes.^5^ However, international research shows that males are more likely to experience severe clinical outcomes, hospitalisation, ICU admittance, and mortality.^4,6,7^

Immunocompromised populations (for example, people receiving cancer therapies such as chemotherapy, radiotherapy, and immunotherapy; and individuals with HIV) appear more likely to contract COVID-19.^8,9^ Comorbidities such as hypertension, diabetes mellitus, cardiovascular diseases, and chronic obstructive pulmonary diseases are also common among those with COVID-19.^10^ To compound matters, individuals with pre-existing health conditions are often more susceptible to severe COVID-19-related illness and injury. At-risk populations include those with weaker immune systems (for example, recipients of immunosuppressant therapy and populations with HIV),^8,9^ and those with chronic health conditions such as hypertension,^10,11^ diabetes,^10,11^ cardiac illnesses,^4,10–12^ chronic lung disease,^13^ liver disease,^14^ haematological malignancy,^15^ chronic kidney disease,^16^ and neurological conditions including dementia, Parkinson’s, epilepsy, multiple sclerosis, and spinal cord injury.^16^ Evidence regarding COVID-19-related health impacts for groups — including pregnant patients and their babies,^17^ patients who are immunocompromised, patients with diabetes,^9,18^ and patients with asthma^19^ — is mixed in terms of clinical outcomes and evidence availability.

Research concerning COVID-19 hospitalisation rates, experiences, and longer-term follow up is varied. Data from the UK and Ireland shows that hospitalisation rates have varied both between countries and as the pandemic has progressed over time.^20,21^ Length of hospital stay, meanwhile, was estimated as 4–53 days in China, with shorter stays (4–21 days) reported elsewhere.^22^ Such discrepancies have been attributed to country-specific differences in admission and discharge criteria.^22^ Reported COVID-19 ICU rates have also varied. In Ireland, ICU rates are estimated to be between 1% and 2%,^5^ while a systematic review of international literature calculated a pooled ICU rate of 15.4%, with 14.9% of patients requiring mechanical ventilation.^23^ Research also suggests that COVID-19 hospital readmission rates are low,^24^ with COVID-19 readmissions most likely to occur among immunocompromised and co-morbid populations.^24,25^ Early rather than later readmissions are more associated with COVID-19 related illness, as opposed to illness arising from pre-existing health conditions.^25^

In the UK, approximately 10% of those diagnosed with COVID-19 require, but do not necessarily receive, care within 3 weeks of infection.^26^ In contrast, research in the US has shown that 35% of people with COVID-19 feel unwell 2–3 weeks following positive diagnosis.^27^ Either way, it is evident that many people recovering from COVID-19 require ongoing care, the kind that is routinely provided in general practice.^26,28^ However, the extent to which those recovering from severe COVID-19 have attended general practice is uncertain. This pilot study sought to address this knowledge gap by investigating the recent GP service use trends of patients with COVID-19 attending a post-COVID-19 follow-up clinic at the Mater Misericordiae University Hospital (MMUH) in Dublin, Ireland.

## Method

This study was conducted and reported according to the STROBE (Strengthening the Reporting of Observational Studies in Epidemiology) guidelines.^29^ The study protocol on which it is based has been reported separately.^30^ This study’s methods deviate somewhat from those outlined in the protocol. For instance, unlike in the protocol, data collection occurred at the MMUH’s post-COVID-19 clinic rather than at general practices in North Dublin. Further, this study had a smaller sample size than was outlined in the protocol, and this study did not examine patients’ COVID-19 clinical outcomes.

### Setting

The study was conducted at the post-COVID-19 follow-up clinic at the MMUH Dublin, Ireland. It is a once-weekly outpatient clinic allowing patients recovering from COVID-19 to be followed up and assessed for persistent COVID-19 symptoms. The patients consist of individuals who have: (a) been hospitalised with COVID-19; (b) been placed on a COVID-19 ambulatory home monitoring programme; and/or (c) been referred from local GPs with COVID-19 infection. Clinical assessment is conducted at the clinic, and relevant diagnostic tests are ordered as clinically indicated (for example, pathology tests, chest X-rays, echocardiography, pulmonary function tests, or computed tomography [CT] of thorax). Patients with persistent symptoms are subsequently referred to a specialist for assessment and treatment.

### Participants

Participants were adult patients seen at the clinic between June and November 2020, and who provided informed consent to participate in the study. Approximately 12–15 patients were offered appointments at the clinic each week. Thus, it is estimated that 250–300 patients were invited to attend the clinic during the entire study period.

### Procedure

Consenting participants completed questionnaires administered by various researcher or healthcare professionals, querying participants’ demographic profiles; prior medical histories; COVID-19 infection details; emergency hospital admissions and readmissions, where applicable; and, where relevant, GP service use trends following hospital discharge.

### Instruments

Participants were asked to indicate their age, sex, and whether they have experienced any of the following conditions: pregnancy, cancer, diabetes, HIV (or other immune-deficiency diseases), heart disease, asthma (requiring medication), other chronic lung diseases, chronic liver disease, haematological disorder, kidney disease, neurological impairment or disease, organ or bone marrow transplant, or ‘other pre-existing condition(s)’. Participants were asked about COVID-19-related hospital experiences, with questions focusing on whether participants were hospitalised, admitted to ICU, and/or readmitted to hospital following an initial stay. Participants were also asked to outline the duration(s) of the above experiences, if relevant. Lastly, where applicable, participants were asked to document whether they had attended GP services within 7 and 30 days of their COVID-19-related hospital discharge.

### Analysis

Data were analysed using descriptive and inferential statistical methods via SPSS (version 26).

## Results

The sample’s (*n* = 153) median age was 43.5 years (IQR = 30.9–52.1 years). There were 105 females (68.6%, 95% CI = 61.3% to 75.9%). Various medical histories were reported among participants, the prevalence of which is outlined below (see Table 1). Sixty-seven participants (43.8%, 95% CI = 35.9% to 51.6%) reported being admitted to hospital with COVID-19. Data outlining the proportion and characteristics of ambulatory home monitoring programme patients, and patients referred to the clinic by GPs were not available.

**Table 1.** Medical history details of patients who were COVID-19 positive (*n* = 153)

**Medical history**	***n* (%**)	**95%** CI
Pregnancy (current)	2^a^(1.3)	0 to 3
Cancer	6 (3.9)	1 to 7
Diabetes	12 (7.8)	3.5 to 12
HIV or other immune-deficiency diseases	0	n/a
Heart disease	10 (6.5)	2.5 to 10.4
Asthma (requiring medication)	13 (8.5)	4 to 12.9
Chronic lung disease (non-asthma)	3 (2)	0 to 4.2
Chronic liver disease	0	n/a
Chronic haematological disorder	2 (1.3)	0 to 3
Chronic kidney disease	1 (0.7)	0 to 2
Chronic neurological impairment or disease	5 (3.3)	0 to 6.1
Organ or bone marrow recipient	1 (0.7)	0 to 2
Other pre-existing condition(s)	100 (65.4)	57.8 to 72.9

a Both second trimester.

### Hospital experiences

Participants’ median hospital stay was 7 days (IQR = 2–15 days). Older individuals were more likely to be hospitalised, *P* ≤0.001 (95% CI = 8.9 to 16.8), as were males, *P* ≤0.001; (φ = -0.283), and people with pre-existing health conditions, *P* ≤0.001; (φ = 0.338), particularly those with heart disease, *P* = 0.005, (φ=0.246). Nine participants (5.9%, 95% CI = 0% to 11.5%) were admitted to ICU, with median ICU stays of 25 days (IQR = 17–37.5 days). Hospitalised patients with chronic lung disease were likely to require ICU care, *P* = 0.045, (φ = 0.338). Seventeen participants (11.1%, 95% CI = 3.5% to 18.6%) had been readmitted to hospital since an initial COVID-19 hospital stay. These patients had a median readmission stay of 3 days (IQR = 1–5.8 days).

### Patients’ GP attendances

Of the 67 participants admitted to hospital, 16 (24%, 95% CI = 13.7% to 34.2%) reported attending a GP within 7 days of hospital discharge, and 26 (39%, 95% CI = 27.3% to 50.7%) reported attending a GP within 30 days of discharge. Data concerning recent GP service usage among the ambulatory home monitoring programme patients, and patients referred to the clinic by GPs were not available.

#### Within 7 days of hospital discharge

The 16 participants attending GPs within 7 days of hospital discharge had a median age of 50.3 years (IQR = 41.7–58.4 years). Eight were male (50%, 95% CI = 25.5% to 74.5%), three had cancer (18.8%, 95% CI = 0% to 37.9%), three had diabetes (18.8%, 95% CI = 0% to 37.9%), three had heart disease (18.8%, 95% CI = 0% to 37.9%), one had chronic lung disease (6.3%, 95% CI = 0% to 18.2%), one had a chronic neurological impairment or disease (6.3%, 95% CI = 0% to 18.2%), and 15 (93.8%, 95% CI = 81.9% to 100%) had ‘other pre-existing condition(s)’. Participants’ median hospital stay was 6.5 days (IQR = 3–14.3 days). Three were admitted to ICU and their ICU stays were 14, 25, and 26 days, respectively. Seven participants (43.8%, 95% CI = 19.4% to 68.1%) were readmitted to hospital since their initial visit. All readmission stays lasted less than 1 week.

#### Within 30 days of hospital discharge

Those attending GPs within 30 days of hospital discharge had a median age of 50.4 years (IQR = 44, 1–57.1 years). This group consisted of 26 patients, of which 13 were males (50%, 95% CI = 30.7% to 69.2%). Five participants (19.2%, 95% CI = 4%to 34.3%) had diabetes, five (19.2%, 95% CI = 4% to 34.3%) had heart disease, three (11.5%, 95% CI = 0% to 23.7%) had cancer, three (11.5%, 95% CI = 0% to 23.7%) had chronic lung disease, one (3.8%, 95% CI = 3.5% to 11.1%) had asthma requiring medication, one (3.8%, 95% CI = 3.5% to 11.1%) had a chronic neurological impairment or disease, and 23 (88.5%, 95% CI = 76.2% to 100%) had an ‘other pre-existing condition'. The median hospital stay for this group was 6 days (IQR = 2.8–15 days). Five participants were admitted to ICU. The median ICU stay was 26 days (IQR = 19.5–58.5 days). Ten participants (38.5%, 95% CI = 19.8% to 57.2%) had been readmitted to hospital since their last COVID-19-related visit, with all readmission stays lasting 3 days or less. When compared to all other responding participants in the sample (that is, both hospitalised and non-hospitalised participants, *n* = 153), those attending GPs within 30 days of hospital discharge were: (a) significantly older, *P* = 0.011 (95% CI = 1.6 to 11.8); (b) more likely to have chronic lung disease, *P* = 0.015 (φ = 0.252); and (c) more likely to have ‘other pre-existing health conditions’, *P* = 0.010 (φ = 0.215).

## Discussion

### Summary

The findings showed that 24% and 39% of patients hospitalised with COVID-19 attended general practice within 7 and 30 days following hospital discharge, respectively. While the findings cannot demonstrate a causal relationship between participants’ GP consultations and COVID-19 health complications requiring follow-up, it is believed it is likely that many of their consultations were COVID-19 related. The authors believe this because the study’s GP attendees possessed characteristics previously linked to persistent COVID-19 complications. For instance, the GP attendees in the sample had an average age of approximately 50 years, several had pre-existing medical conditions, and several others had been admitted to ICU, and/or were readmitted to hospital with further COVID-19-related health complications.

### Strengths and limitations

The COVID-19 clinic at the MMUH, Dublin, and the clinical staff working there, played an instrumental role with regards to recruiting and collecting data from this study’s participants. The clinic facilitated routine access to a hard-to-reach population during a period of strict COVID-19 restrictions on movement in Ireland, and so its contribution to this study cannot be underestimated. Further, the study’s reporting process benefited from adopting the STROBE guidelines for observational studies,^29^ and from guidelines outlined in the study protocol.^30^ The generalisability of this study’s findings, meanwhile, are limited by the smaller size of the sample, particularly the GP attendee samples, and by the fact that convenience rather than random sampling techniques were used for feasibility purposes. Also, participants were not questioned as to whether their GP visits were COVID-19 related. Thus, the findings could not demonstrate a causal relationship between COVID-19 illness and general practice attendances. Lastly, the authors did not have data outlining the recent GP service use trends of patients in the sample who were not hospitalised (that is, those placed on the COVID-19 ambulatory home monitoring programme, and those referred to the clinic from local GPs). It is unclear whether these groups’ GP service usage trends would have differed from those of the hospitalised group.

### Comparison with existing literature

The finding that 24% and 39% of patients hospitalised with COVID-19 require follow-up care contrasts with estimates for all individuals diagnosed COVID-19 with COVID-19 from the UK (10%)^26^ and the US (35%).^27^ The findings also provide support for claims that older adults^3^ and individuals with pre-existing health conditions^9–13^ are more likely to experience ongoing COVID-19 complications requiring follow-up. Moreover, it was notable that this study’s GP attendees had higher readmission rates than those observed among the general population in previous studies.^24,25^

### Implications for research and practice

This pilot study’s findings suggest that persistent COVID-19 complications could be a serious public health concern. Thus, larger-scale studies are necessary to obtain more precise estimates of persistent illness following severe COVID-19 cases in the general population(s). It is recommended that such studies consult the Method and Strengths and limitations sections of this article for guidance on how to design their research. Qualitative research and studies comparing the prevalence of persistent COVID-19 illness between countries with varying socioeconomic profiles, health indexes, COVID-19 response strategies, and healthcare systems may also be informative. Meanwhile, the authors acknowledge that this was a pilot study with a small sample, and that any recommendations that are made for clinical practice must be done cautiously. With this in mind, the authors simply ask that clinicians be mindful of persistent COVID-19 health issues in the community, vulnerable groups that may be more susceptible to persistent COVID-19 concerns, and relevant ongoing research activities and findings.

In conclusion, this pilot study’s findings suggest that persistent health concerns requiring ongoing care may be common among people with severe COVID-19 infection. The findings also suggest that among populations with severe COVID-19, older adults, individuals with pre-existing medical conditions, and those requiring ICU and/or hospital readmission may have greater ongoing care needs. Large scale studies are required to gain more precise estimates of persistent illness among severe COVID-19 cases in the general population(s).
